# Evaluation of Health Care Providers Satisfaction with the Implementation of a Transitional Pain Service

**DOI:** 10.3390/jcm12020537

**Published:** 2023-01-09

**Authors:** Manouk Admiraal, Jeroen Hermanides, Markus W. Hollmann, Henning Hermanns

**Affiliations:** Department of Anesthesiology, Amsterdam University Medical Center, University of Amsterdam, Meibergdreef 9, 1105 AZ Amsterdam, The Netherlands

**Keywords:** transitional pain service, health care providers, satisfaction

## Abstract

Chronic postsurgical pain develops in 10% of patients undergoing surgery. Recently, multidisciplinary, patient-tailored interventions, such as a Transitional Pain Service (TPS) have been developed and implemented to improve perioperative pain management and thereby prevent chronic postsurgical pain. The purpose of this survey was to analyse health care providers satisfaction and learn from their experiences on the implementation of a TPS. In the TRUST study, a randomized controlled trial investigating the effectiveness of a TPS, 176 patients were enrolled. Afterwards, a satisfaction survey was internally developed, which consisted of eight items. Satisfaction was measured using a Likert scale with five response options from never (1 point) to always (5 points). Surveys were sent to all anaesthetists and anaesthesia residents in our department that were faced with the consequences of TPS implementation. In May 2022, 36 caregivers of the Department of Anaesthesiology returned the survey after four rounds of distribution, with a response rate of 82.3%. Thirty staff members (81.0%) strongly felt that patient care had improved with the introduction of a TPS and 33 (86.8%) would like to see the TPS to be continued in the future. Health care provider satisfaction improved after implementation of a TPS in our hospital.

## 1. Introduction

Chronic postsurgical pain (CPSP) develops in 10% of patients undergoing surgery [1]. Since more than 320 million people undergo surgery each year, this is a major challenge [2]. Severe and undertreated acute pain after surgery can result in delayed recovery, chronic postsurgical pain (CPSP) and chronic opioid use [3]. Despite at least 110 double-blinded, placebo-controlled, randomized studies assessing perioperative administered drugs to prevent CPSP, little progress has been made in recent decades [4]. More pragmatic, multidisciplinary, patient-tailored approaches, such as a Transitional Pain Service (TPS), are now being developed and implemented to enhance perioperative pain management and thereby prevent CPSP [5]. Recently, we investigated the effectiveness of a TPS in a randomized controlled trial (RCT), the TRUST study [6]. For a successful implementation of a new intervention and optimal protocol adherence, care provider satisfaction is essential. Several studies showed that physician job satisfaction may influence quality of care and patient relationships [7], while lack of acceptance among care providers may lead to non-compliance and, hence, suboptimal practice. Staff satisfaction during implementation of a multidisciplinary TPS has not been investigated yet. The purpose of this survey was to analyse several aspects of health care providers satisfaction, such as communication, work-load, and effectiveness, and learn from their experiences on the implementation of a TPS.

## 2. Materials and Methods

In the TRUST study, a TPS was implemented and its effectiveness was studied in patients at increased risk of CPSP who underwent surgery. The accredited medical research ethics committee of the Academic Medical Centre in Amsterdam (2020_211) obtained ethics approval on 15 October 2020. For the survey amongst care providers, the Medical Research Involving Human Subjects Act (WMO) did not apply because the survey did impose actions or rules on the subjects. We adhered to the General Data Protection Regulation of the European Union. The trial is registered with the Netherlands Trial Register, NL9115. Before implementation of the TPS, standard of care (SOC) concerning postoperative pain treatment at the Amsterdam UMC included the Acute Pain Service (APS), which is nurse-based and anaesthetist supervised. This SOC group served as control group within the TRUST trial. For a complete outline of the study design and detailed overview on our TPS, a study protocol was published [6].

TPS was designed to identify patients whose perioperative pain management was expected to be more complex and who were at risk of developing CPSP after surgery. A multidisciplinary team of clinicians provided care, starting before surgery until months after discharge from the hospital. A patient-tailored, pharmacological and/or interventional pain management plan was discussed within the team and modified as necessary in the months that followed. In addition to reducing acute pain and transition to chronic pain, the TPS assists in tapering opioids when necessary, improving coping and functioning despite remaining pain. Before implementation of the TPS, we have put up posters with information about the TPS in various places in our department. These posters included information about TPS referrals with phone numbers of TPS members attached.

The TPS care provider satisfaction survey was an internally developed instrument to measure care provider satisfaction with the implementation of a TPS, which consisted of eight items. The selection of items was based on discussion between the authors and assuming a 1:5 item to subject ratio with an expected inclusion of approximately 40 subjects at our department. The survey questions were selected to cover all aspects the authors could think of that were relevant to evaluate the TPS. First, MA proposed a set of questions to the TPS team, which were complemented, resulting in a new set of items. These were subsequently reviewed, improved, and complemented by the HH and JH. The new set of questions was then reviewed by MWH, after which a final version was achieved at. 

The questions covered accessibility, communication, times savings, feasibility, improvement in care, loss of control, and future existence. Satisfaction was measured using a Likert scale with five response options from never (1 point) to always (5 points). Additional comments in free text could be placed at the bottom of the form. Surveys were sent to all anaesthetists and anaesthesia residents in our department, that were faced with the consequences of TPS implementation within 3 months after the last patient finished the short-term follow-up. Data were collected anonymously using Google Forms (Alphabet Co., Mountain View, CA, USA), and a descriptive analysis was performed. A subgroup analysis, applying a Kruskal–Wallis test was performed (IBM SPSS Statistics, Version 28.01.1, Chicago, IL, USA), exploring any differences in responses between anaesthetists and anaesthesia residents.

## 3. Results

**Figure 1:**
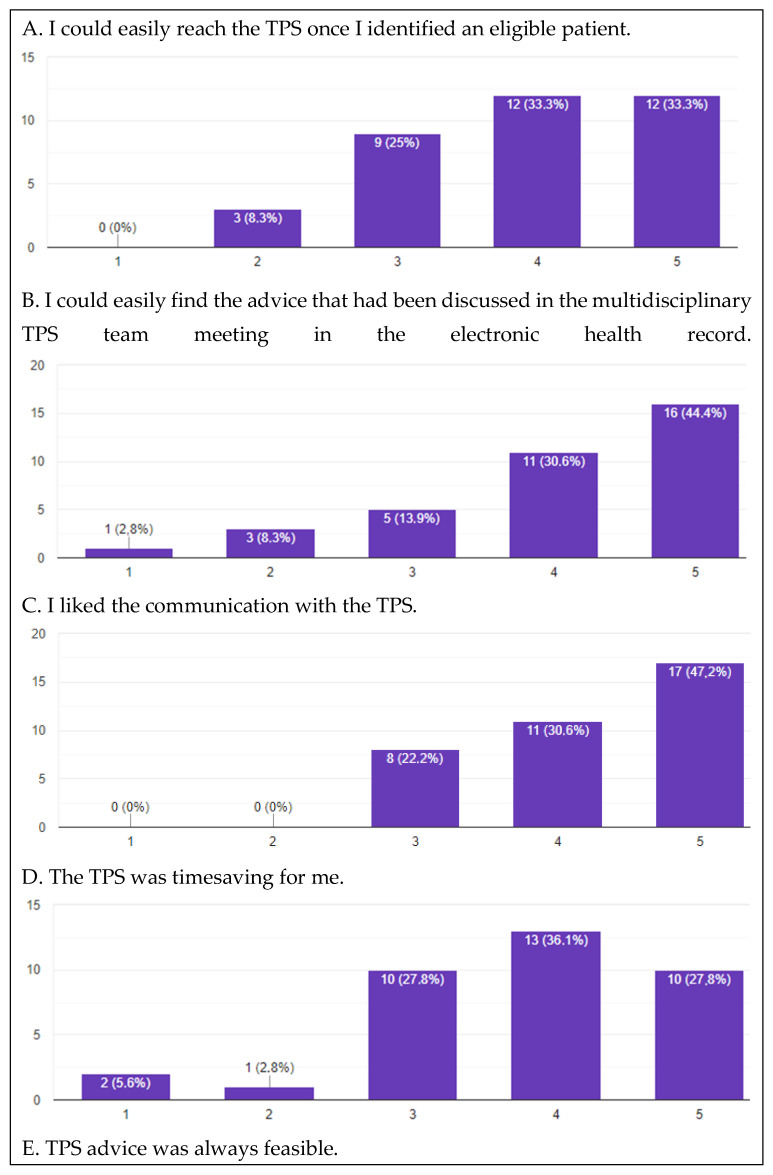

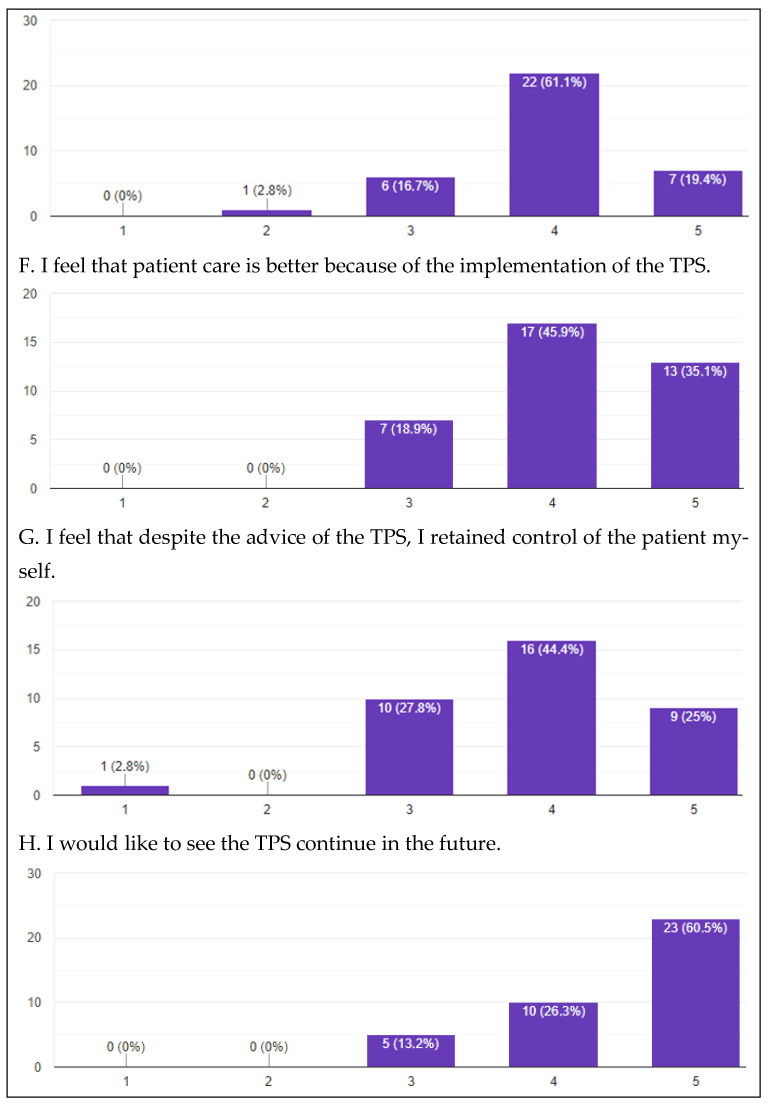
Bar charts answers of care providers satisfaction in the Transitional Pain Service survey. Satisfaction was measured using a Likert scale including a range of five response options from never (1 point) to always (5 points). Abbreviations: TPS = Transitional Pain Service.

In May 2022, a total of 36 caregivers of the Department of Anaesthesiology returned the survey after four rounds of distribution. The survey response rate was 82.3%, however, two staff members have not fully completed the questionnaire. Satisfaction with the TPS was depicted in Figure 1. Regarding accessibility, three (8.3%) responders could not easily reach the TPS once an eligible patient was identified. In addition, four (11.1%) of staff members could not easily retrieve the advice discussed during the multidisciplinary TPS meeting. However, 28 (84.1%) responders were satisfied regarding the communication with the TPS team. Then, 29 (80.5%) felt that the advice given by the TPS was feasible, and 23 (63.9%) staff members felt the TPS was time-saving. Furthermore, 30 staff members (81.0%) strongly felt that patient care had improved with the introduction of a TPS and 33 (86.8%) would like to see the TPS be continued in the future. Additional comments in free text are displayed in Table 1. In general, comments included that the TPS was a valuable additional service. One respondent suggested expanding the service to non-surgical wards. In addition, one respondent regarded the TPS recommendations as “a bit too general” as for example multi-modal pain therapy is already applied for many types of surgery. Finally, the subgroup analysis exploring differences in responds between anaesthetists and anaesthesia residents yielded no significant differences.

## 4. Discussion

With the ongoing opioid pandemic, new approaches are being developed to prevent CPSP and chronic opioid use. After performing the first RCT studying the TPS for short-term outcomes, we also evaluated caregiver satisfaction and found that caregivers were generally highly satisfied with the implementation of TPS and like to see it be continued and extended in the future. The comment that some recommendations were a bit “too general”: e.g., multi-modal pain policy, something we generally always apply to major abdominal surgery has been reported back to the TPS for the implementation, although this may also reflect the study effect, i.e., the introduction of the TPS may have improved the peri-operative pain care in general. We found no differences between anaesthetists and anaesthesia residents, which is unsurprising, since both groups work in the same way with the TPS. In addition, our study was not powered for this specific analysis.

Our study has its inherent limitations of a small sample size in a single centre, and the use of a newly developed questionnaire, however we believe to have covered most aspects of care provider satisfaction when implementing a TPS. In addition, this is, to our knowledge, the first evaluation of care provider satisfaction in this context.

Already in 2016, Huang et al. suggested implementing a TPS after reporting high rates of CPSP and chronic opioid use after surgery [8]. Around that time, several uncontrolled cohort studies published promising results after implementing a TPS, such as a decrease in opioid use [9,10]. In agreement with our findings, Buys et al. reported improved satisfaction among health care providers with the implementation of a TPS, emphasizing that this improvement in multidisciplinary TPS teams constitutes one significant outcome of TPS [11]. We argue that these effects, in turn, are of benefit, especially for high-risk patients, such as those with chronic opioid abuse.

To further enhance communication on TPS in the future, we will incorporate a tool in the electronic patients’ record providing a direct referral to the TPS from the pre-operative screening record. Furthermore, if a patient is treated by the TPS a memo will be added in the patient’s record to improve the discoverability of the recommendations.

The limitations of our survey include assessment in a single centre only, owing to the fact that TPS was initially implemented at our centre first. We hope that other hospitals will follow in the future. Furthermore, partly, there is some small overlap between investigators and participants of the survey (*n* = 2). Finally, there might be more elaborate tools to measure satisfaction than the Likert scale—there is, however, no evidence showing that those are superior. Given that the Likert Scale is the most universal method for survey collection, it can easily be understood, and the responses are readily quantifiable.

In conclusion, health care provider satisfaction improved after implementation of a TPS in our hospital.

## Figures and Tables

**Table 1:** Additional comments in free text survey, translated to English from Dutch.

Perhaps also extend to patients who are already clinical, e.g., also to the internal medicine, etc.
Useful addition to existing practice. Knowledge and skills regarding complex pain patients are generally insufficient to make good perioperative plans for this group.
The TPS is an additional service on what I think is a good basic package.
Great service that is really needed.
Very valuable!
Sometimes I found the recommendations a bit “too general”: e.g., multi-modal pain policy, something we generally always apply to major abdominal surgery…

## Data Availability

The data that support the findings of this study are available from the corresponding author upon reasonable request.
